# Effect of Computerized Physician Order Entry Reminders on HLA-B*15:02 Screening Rates: A Retrospective Study in a Taiwanese Hospital

**DOI:** 10.3390/healthcare13161941

**Published:** 2025-08-08

**Authors:** Xiao Chen, Jason Jiunshiou Lee, Mei-Hsiouh Guan, Su-Han Hsu, Shu-Chuan Wu

**Affiliations:** 1 Department of Pharmacy, Taipei City Hospital, Taipei 108, Taiwan; a5618@tpech.gov.tw; 2 Department of Family Medicine, Taipei City Hospital Yangming Branch, Taipei 111, Taiwan; dak50@tpech.gov.tw; 3 Department of Health and Welfare, University of Taipei, Taipei 100, Taiwan; 4 Institute of Public Health, National Yang Ming Chiao Tung University, Taipei 112, Taiwan; 5 Department of Health Care Management, National Taipei University of Nursing and Health Sciences, Taipei 112, Taiwan; 6 Department of Pharmacy, Taipei City Hospital Zhongxiao Branch, Taipei 115, Taiwan; a0697@tpech.gov.tw; 7 Department of Pharmacy, Taipei City Hospital Yangming Branch, Taipei 111, Taiwan; 8 School of Pharmacy, College of Pharmacy, Taipei Medical University, Taipei 110, Taiwan; 9 Department of Exercise and Health Sciences, University of Taipei, Taipei 111, Taiwan

**Keywords:** HLA-B*15:02, carbamazepine, CPOE reminders, pharmacogenomics

## Abstract

**Background:** Carbamazepine (CBZ) is associated with severe cutaneous adverse reactions in individuals carrying the HLA-B*15:02 allele, which is prevalent in Asian populations. Genetic screening before the initiation of CBZ is recommended, yet screening is not always undertaken. **Objectives:** To determine the effect of implementing computerized physician order entry (CPOE) reminders on the screening rates of HLA-B*15:02 before CBZ prescription. **Methods:** We conducted a retrospective analysis of 1611 patients who were prescribed CBZ between 2012 and 2023 in a regional hospital in Taiwan. The intervention involved integrating automated HLA-B*15:02 screening reminders into the CPOE system in outpatient settings. Patients were divided into an outpatient (intervention) group and an inpatient (control) group, and their data were analyzed before and after the intervention. Screening rates were compared using Fisher’s exact test, and subgroup analyses were conducted on the basis of age group and physician specialty. **Results:** After the intervention, the outpatient group exhibited an increase in screening rate from 23.7% to 55.6% (*p* < 0.001). However, no significant change was observed in the inpatient group. Subgroup analysis revealed major improvements among neurologists and patients aged 41–80 years in outpatient settings. **Conclusions:** Implementing CPOE reminders substantially improves rates of screening for HLA-B*15:02 in outpatient settings, indicating the effectiveness of informatic interventions in enhancing adherence to pharmacogenomic guidelines. Extending such interventions to inpatient settings may further mitigate the risk of CBZ-induced severe cutaneous adverse reactions.

## 1. Introduction

Carbamazepine (CBZ) is a medication used in neurology for the management of epilepsy and trigeminal neuralgia with the ability to control seizures and relieve neuropathic pain. Despite its clinical utility, CBZ is associated with severe cutaneous adverse reactions (SCARs), including Stevens–Johnson syndrome (SJS) and toxic epidermal necrolysis (TEN) [1]. Although these dermatological conditions are rare, they are potentially life-threatening, with TEN patients having mortality rates of up to 30%. These severe outcomes necessitate the early identification of high-risk individuals to prevent catastrophic complications during CBZ therapy [2].

In Taiwan, SCARs have been a primary cause of drug injury relief cases, accounting for 67.35% of all such cases reported between 1999 and 2023, according to data from the Taiwan Drug Relief Foundation. Although CBZ was historically the fourth most commonly implicated drug, its ranking dropped to tenth between 2018 and 2023. This decline likely reflects increased awareness of its risks and the growing use of alternative medications. Nevertheless, CBZ-induced SCARs remain a major clinical concern because of their preventable nature and substantial morbidity and mortality [3].

A key contributing factor is genetic susceptibility. The HLA-B*15:02 allele, which is strongly associated with CBZ-induced SCARs, has a high prevalence in Asian populations. Reported allele frequencies include 11.6% in Singapore, 10.2% among Han Chinese, and 5.3% to 8.4% in other Asian regions [4], while frequencies are negligible in Japan [5,6] and Korea [7]. Multiple studies conducted across Asia, including in Hong Kong [8], Thailand [9,10], India [11,12], Malaysia [13], and Vietnam [14], have confirmed this association.

In Taiwan, the incidence of CBZ-induced SJS and TEN is estimated to be 59 cases per 100,000 new users annually, which is markedly higher than the incidence of 2 cases per 100,000 new users reported in the United States [15]. This disparity is attributable to the prevalence of the HLA-B*15:02 allele, a genetic variant strongly associated with CBZ-induced SCARs.

In Taiwan, the link between HLA-B*15:02 and CBZ-induced SJS is particularly robust. Chung et al. [16] reported that all patients with CBZ-induced SJS had the HLA-B*15:02 allele, with a 2504-fold higher risk compared with that faced by CBZ-tolerant individuals. Subsequent investigations confirmed these findings, revealing a 1357-fold increased risk among HLA-B*15:02 carriers. Overall, these findings underscore the importance of integrating pharmacogenomic testing into prescription practices to mitigate the risk of severe adverse reactions [17].

In response to the accumulating evidence linking HLA-B*15:02 to CBZ-induced SCARs, both international and national regulatory agencies introduced targeted safety policies. In 2007, the United States Food and Drug Administration issued a recommendation for the genetic screening of HLA-B*15:02 in Asian patients before the initiation of CBZ therapy [4]. In Taiwan, the National Health Insurance Administration implemented a reimbursement program for HLA-B15:02 testing in 2010, aimed at enhancing pharmacovigilance and reducing preventable adverse drug reactions [18]. These policies highlight the global recognition of pharmacogenomics in personalized medicine; however, real-world implementation has remained inconsistent—largely due to gaps in physician awareness and the lack of integrated decision-support tools. In many clinical settings, gaps in physician awareness, time constraints, and the absence of automated clinical decision support have limited adherence to these guidelines.

To address the aforementioned limitations, our hospital implemented a computerized physician order entry (CPOE) system with the genetic testing reminder feature launched on 1 September 2022, integrating automated reminders for HLA-B*15:02 screening into routine clinical workflows. This intervention aimed to close existing gaps in clinical implementation, promote adherence to pharmacogenomic guidelines, and improve patient safety.

Therefore, the objective of this study was to evaluate the impact of CPOE reminders on HLA-B*15:02 screening rates among new CBZ users in a regional Taiwanese hospital.

## 2. Materials and Methods

### 2.1. Data Sources

Clinical and prescription data were extracted from the clinical database of a regional hospital in Taipei, Taiwan that has eight branches distributed throughout the city. Because SJS and TEN predominantly occur within the first 2 months after the initiation of CBZ therapy [19], we defined the acceptable time frame for HLA-B*15:02 genetic testing as within 2 months after the initiation of CBZ therapy. Specifically, patients could undergo HLA-B*15:02 testing either before or within 2 months after being prescribed CBZ. Beyond this time frame, HLA-B*15:02 testing data were excluded from the analysis.

Prescription data were collected from July 2011 to August 2023, and HLA-B*15:02 genetic testing data were collected from July 2011 to October 2023. This study was approved by the Institutional Review Board of Taipei City Hospital (approval no. TCHIRB-11305011-E).

### 2.2. Study Design and Population

This retrospective study included adult patients who initiated CBZ therapy between January 2012 and August 2023. This retrospective study included CBZ-treated patients who had at least a single outpatient department visit, emergency room visit, or hospitalization event between July 2011 and August 2023. To ensure the inclusion of actual new users of CBZ, only patients who began CBZ therapy after January 2012 were included in the analysis. Patients aged younger than 18 years were excluded.

The CPOE reminder was implemented as a pop-up alert that appeared when a prescriber attempted to order CBZ for a patient without a documented HLA-B*15:02 test result. The alert recommended ordering the test before proceeding. This was designed as a soft stop: the physician could override the alert and continue prescribing if clinically justified.

The study cohort was divided into two groups on the basis of treatment setting. The intervention (outpatient) group included patients who were prescribed CBZ in outpatient department or emergency room settings with HLA-B*15:02 genetic testing reminders. The control (inpatient) group included patients who were prescribed CBZ during hospitalization with no genetic testing reminders. Each group was further subdivided into two groups on the basis of intervention timeline:

Preintervention period: patients who were prescribed CBZ from January 2012 to August 2022.

Postintervention period: patients who were prescribed CBZ from September 2022 to August 2023 after the introduction of genetic testing reminders on 1 September 2022.

This stratification facilitated a comparative analysis of the effect of CPOE-integrated reminders on HLA-B*15:02 genetic testing rates across different clinical settings and time frames.

### 2.3. Statistical Analysis

Statistical analyses were conducted to evaluate changes in HLA-B*15:02 screening rates before and after the intervention across both outpatient and inpatient groups. Annual data on the number of new CBZ users and HLA-B*15:02 genetic testing rates were collected and analyzed from 2012 to 2023. The year 2022 was divided into two segments: from 1 January to 31 August 2022 and from 1 September to 31 December 2022.

Patient characteristics (e.g., sex, age, and treating physician’s specialty) and details of the first CBZ prescription (e.g., primary diagnosis) were collected for each participant. All statistical analyses were conducted using IBM SPSS Statistics version 21 (IBM, Armonk, NY, USA). Descriptive analyses were used to summarize annual screening trends.

Fisher’s exact test was used to compare the screening rates of HLA-B*15:02 before and after the intervention in the two groups. The test was selected due to the relatively small sample size in some post-intervention subgroups, where expected cell counts may have been too low to meet the assumptions of the chi-square test. Screening rates were analyzed by age and physician specialty to determine their association with testing rates. A two-sided *p*-value of < 0.05 was considered statistically significant.

## 3. Results

### 3.1. Patient Characteristics

**Figure 1:**
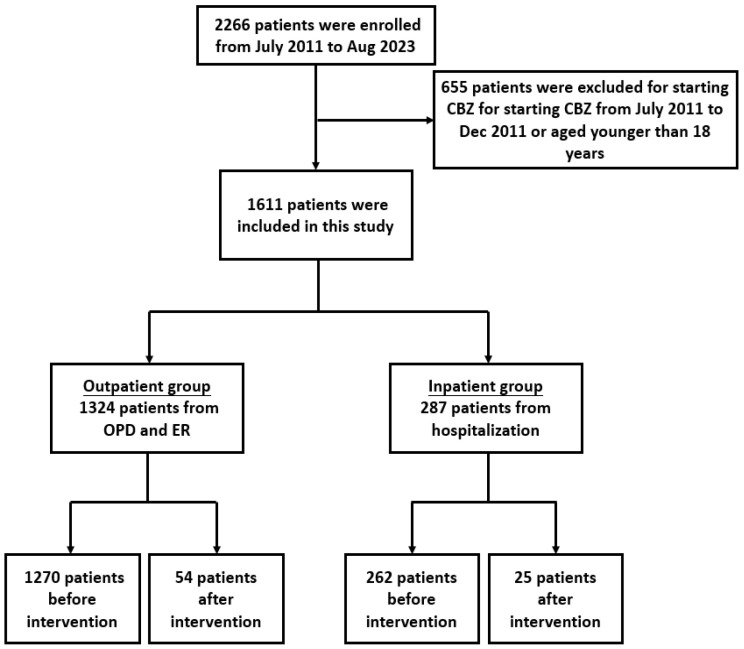
Patient inclusion process.

A total of 2266 patients were initially identified. Of these patients, 655 who were started on CBZ therapy before January 2012 were excluded. In total, 1611 patients were included in the final analysis (Figure 1). Of these patients, 1324 were categorized into the outpatient group (treated in outpatient department or emergency room settings), and 287 were categorized into the inpatient group (treated during hospitalization).

In the outpatient group, 1270 patients received CBZ before the intervention, and 54 patients received CBZ after the intervention. Similarly, in the inpatient group, 262 patients received CBZ before the intervention, and 25 patients received CBZ after the intervention. Notably, the outpatient group (n = 1324) was substantially larger than the inpatient group (n = 287), resulting in an imbalance in sample size. This discrepancy may have implications for statistical power, particularly in subgroup analyses and between-group comparisons.

In the outpatient group, 57.5% were women, with a mean age of 55.5 ± 18.3 years, and 33.9% were in the 61–80-year age group. Neurology was the most common physician specialty (31.5%), with nearly half of the indications for CBZ (47.2%) being off-label. In the inpatient group, 50.5% of the patients were women, with a mean age of 55.9 ± 18.9 years, and 31.7% were also in the 61–80-year age group. Psychiatry was the most common physician specialty (38.0%), with the majority of the indications for CBZ (77.7%) being off-label (Table 1).

Patient characteristics were analyzed before and after the intervention in the outpatient and inpatient groups. In the outpatient group before the intervention, 57.4% were women, with a mean age of 55.6 ± 18.2 years, and 33.9% were in the 61–80-year age group. Neurology physicians treated 30.7% of the patients, and 47.3% of CBZ prescriptions were for off-label use. In the outpatient group after the intervention, 59.3% were women, with a mean age of 54.5 ± 19.6 years, and 33.3% were in the 61–80-year age group. Neurology physicians treated 50.0% of the patients, and 44.4% of CBZ prescriptions were for off-label use.

In the inpatient group before the intervention, 50.8% of the patients were women, with a mean age of 56.4 ± 18.9 years, and the largest proportion (32.1%) were in the 61–80 age group. Psychiatry physicians treated 37.4% of the patients, and 77.9% of CBZ prescriptions were for off-label use. After the intervention, 52.0% of the patients were women, with a mean age of 49.9 ± 18.4 years, and 41–60 years each accounted for 32.0%. Psychiatry physicians treated 44.0% of the patients, and 76.0% of CBZ prescriptions were for off-label use.

### 3.2. CBZ Usage Trends

**Figure 2:**
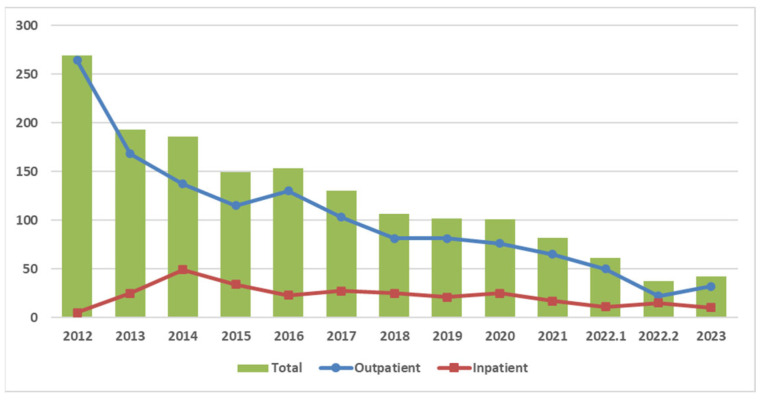
CBZ usage trends. Notes: 1. The year 2022 was split into 1 (January–August 2022) and 2 (September–December 2022) based on the intervention date of 1 September 2022. 2. The CPOE genetic testing reminder was launched on 1 September 2022, and applied only to outpatient settings.

From 2012 to 2023, 1611 patients were prescribed CBZ. The number of new users peaked in 2012 and then steadily declined. A notable reduction in the number of new users was observed after 2016 (Figure 2).

### 3.3. Trends in HLA-B*15:02 Screening Rates and Factors Associated with Screening

**Figure 3:**
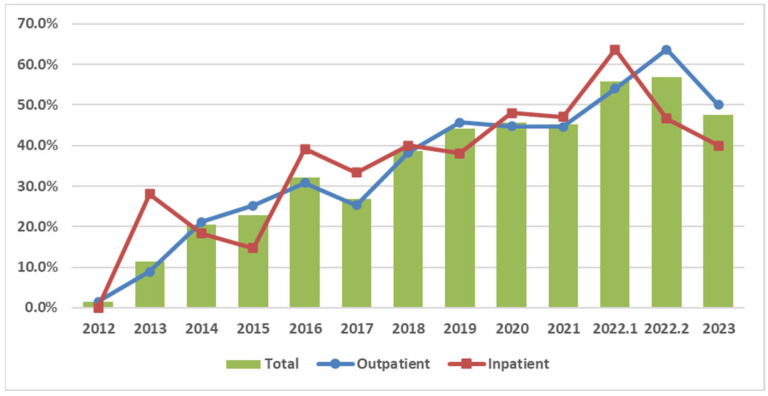
Trends in HLA-B*15:02 screening rates by group. Notes: 1. The year 2022 was split into 1 (January–August 2022) and 2 (September–December 2022) based on the intervention date of 1 September 2022. 2. The CPOE genetic testing reminder was launched on 1 September 2022, and applied only to outpatient settings.

The HLA-B*15:02 screening rates increased over time. In the outpatient group, the screening rates reached their peak after the intervention, whereas in the inpatient group they did not (Figure 3). This contrast visually underscores the effectiveness of the CPOE reminder system implemented exclusively in outpatient settings.

**Figure 4:**
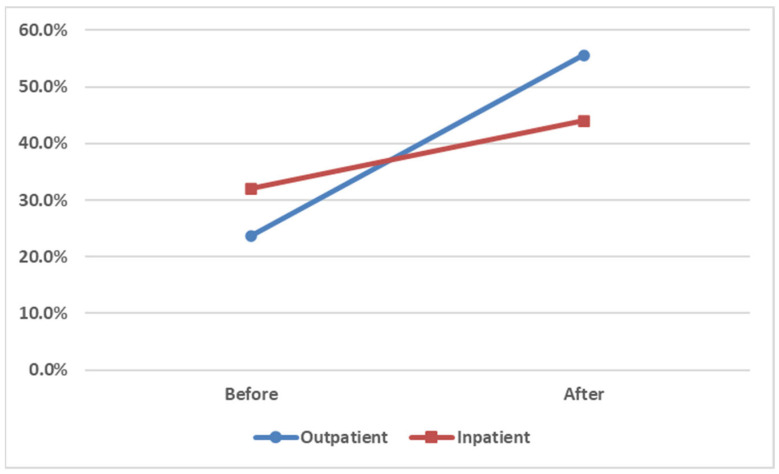
Comparison of HLA-B*15:02 screening rates before and after the intervention in the outpatient and inpatient groups.

After the CPOE intervention, the screening rates of the outpatient group significantly increased from 23.7% to 55.6% (*p* < 0.001). This substantial increase in screening uptake suggests that CPOE-based alerts may serve as an effective and scalable strategy to promote pharmacogenomic guideline adherence in busy outpatient settings. However, since there were no genetic testing reminders for the inpatient group, its screening rates did not significantly increase (*p* = 0.225). These results indicated that the intervention significantly increased the screening rates. Screening rates increased significantly in the outpatient group (OR = 4.02; 95% CI: 2.31–6.98; *p* < 0.001). (Figure 4 and Table 2) This evidence supports the hypothesis that system-level prompts significantly enhance pharmacogenomic testing adherence.

**Figure 5:**
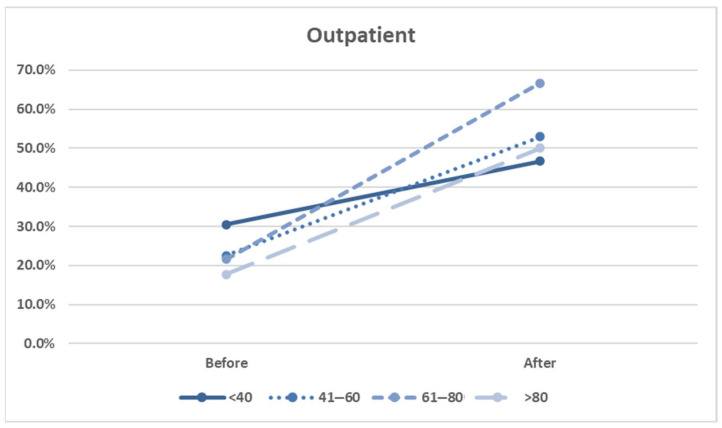
Factors associated with HLA-B*15:02 screening rates in outpatients, stratified by age group.

Our age group analysis revealed the following. In the outpatient group, the screening rates significantly increased for patients aged 41–60 years (*p* = 0.004) and 61–80 years (*p* < 0.001) after the intervention (Figure 5 and Table 3). These findings suggest that physicians may perceive middle-aged and older patients as being at greater risk or more likely to benefit from pharmacogenomic guidance.

**Figure 6:**
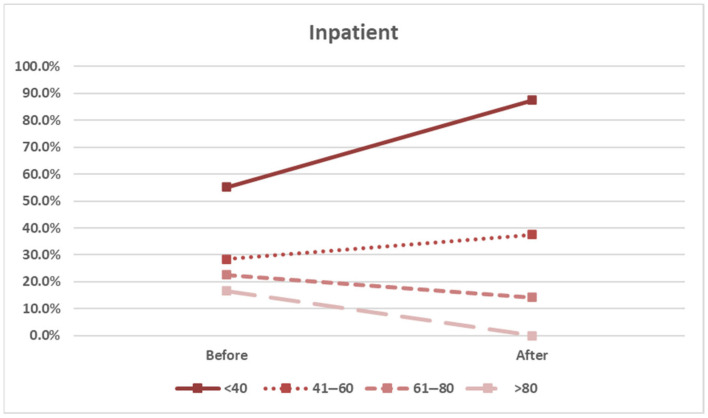
Factors associated with HLA-B*15:02 screening rates in inpatients, stratified by age group.

However, in the inpatient group, the screening rates slightly decreased for patients aged over 60 years (Figure 6 and Table 4). This finding may point to systemic limitations in inpatient workflows or prioritization issues that warrant targeted policy or educational intervention.

**Figure 7:**
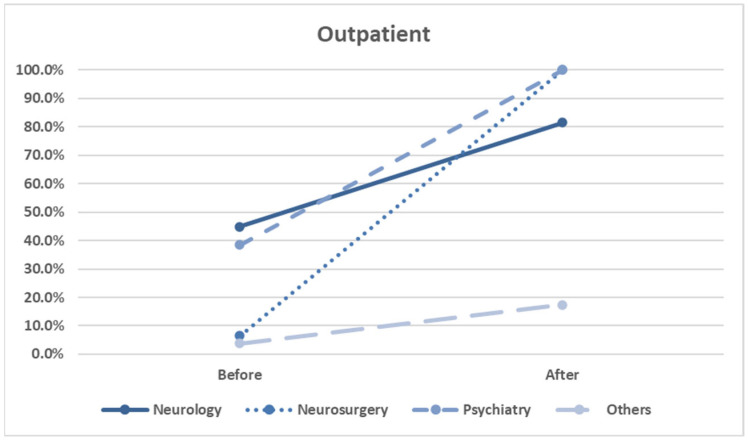
Factors associated with HLA-B*15:02 screening rates of outpatients, stratified by physician specialty.

Our physician specialty analysis revealed the following. After the intervention, a significant increase in screening rates was observed among outpatient physicians specializing in neurology (*p* < 0.001), neurosurgery (*p* < 0.001), and other specialties (*p* = 0.002). (Figure 7 and Table 5). This suggests that the CPOE reminders were most effective when aligned with physicians who regularly manage CBZ-related conditions.

**Figure 8:**
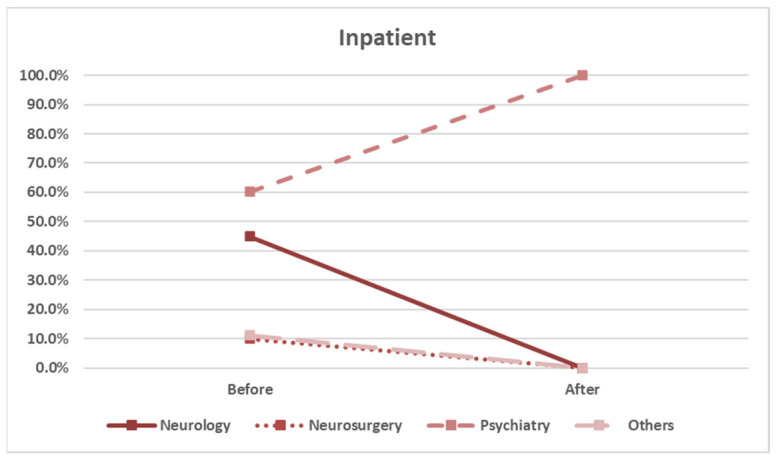
Factors associated with HLA-B*15:02 screening rates of inpatients, stratified by physician specialty.

In the inpatient group, the screening rates significantly increased only among psychiatry specialists (*p* = 0.009), whereas the screening rates for neurology and other specialties decreased (Figure 8 and Table 6). This may reflect spillover effects from outpatient settings or institutional practices in psychiatric branches.

Multivariable logistic regression revealed that the effect of the CPOE reminder was largely driven by outpatient prescribing (interaction OR = 4.20; 95% CI: 1.20–14.74; *p* = 0.021), while intervention alone was not a significant predictor (OR = 1.58; *p* = 0.260). Physician specialty and diagnosis also significantly influenced screening behavior, with neurology and psychiatry showing markedly higher adherence compared to other specialties. These subgroup differences underline the need for targeted education or workflow adjustments, particularly among departments or physician groups with persistently low screening rates (Table 7).

## 4. Discussion

In this retrospective study, we demonstrated a significant reduction in the number of new CBZ users admitted to our hospital from 269 in 2012 to 42 in 2023. This trend is mirrored by the findings of Chang et al. [20], who demonstrated a nationwide decline in CBZ use across Taiwan. This decline is likely attributable to the introduction of new antiepileptic and neuralgia medications, such as pregabalin and levetiracetam. It is expected to be driven by the emergence of alternative antiepileptic and neuralgia medications (e.g., pregabalin, levetiracetam) that offer comparable efficacy with lower SCARs risk. These prescribing shifts reflect a growing emphasis on drug safety and the clinical integration of pharmacovigilance principles.

Overall, our HLA-B*15:02 screening rates are lower than those reported by other regional hospitals in 2014 (e.g., 24.9% overall and 51.4% for regional hospitals) [21]. Even after the CPOE intervention, 44.4% of patients still received CBZ without undergoing HLA-B15:02 screening beforehand, suggesting persistent barriers in clinical uptake. While the CPOE reminder led to improvement, nearly half of the eligible patients still did not undergo testing before CBZ initiation. This highlights practical challenges such as physicians’ unfamiliarity with the CPOE system and the absence of a punishment or reward mechanism, which led to physicians not paying attention to the CPOE, or patients’ reluctance to undergo HLA-B15:02 screening due to concerns about additional blood draws or long waiting times. However, our screening rates are higher than those reported in 2017 (16%) [20]. This discrepancy may be attributable to differences in hospital practices, physician training, or patient demographics. These variations underscore the importance of tailored interventions to address local barriers to pharmacogenomic testing.

Implementing CPOE-based reminders in outpatient settings increased the screening rates of HLA-B*15:02 from 23.7% to 55.6% (*p* < 0.001), indicating the effectiveness of technology-driven interventions in promoting adherence to pharmacogenomic guidelines. The absence of a similar intervention in inpatient settings may explain the disparity observed in screening rates between these two settings. This supports the role of health IT in reducing preventable adverse drug reactions and aligning clinical practice with precision medicine guidelines. Before the intervention, physicians in outpatient settings may have been less familiar with the clinical importance of genetic testing before prescribing CBZ. These reminders served as an essential educational tool that prompted physicians to prioritize testing, thereby enhancing patient safety.

Interestingly, a modest increase in screening was observed among psychiatric inpatients, despite the absence of direct CPOE alerts. This may reflect a spillover effect from the outpatient setting—particularly in branches with integrated psychiatric services. Furthermore, some physicians cared for both outpatient and inpatient populations, and their exposure to reminders may have indirectly influenced testing behavior in inpatient contexts. Physicians exposed to CPOE reminders in the outpatient setting may have been more inclined to order genetic testing for inpatients as well. Nevertheless, the overall inpatient screening trend remained statistically nonsignificant, underscoring the need for targeted system-level interventions tailored specifically to inpatient workflows.

Our findings are consistent with those of a study reporting higher screening rates in younger patients [21]. After the intervention, the screening rates increased across all age groups in the outpatient settings, although older patients (>60 years) in the inpatient group exhibited a slight decline. This trend may reflect implicit biases or clinical prioritization patterns, wherein older patients are perceived to benefit less from pharmacogenomic testing due to shorter treatment horizons or acute care demands. These findings point to the importance of age-sensitive clinical decision-making support.

Subgroup analysis by specialty revealed that the screening rates were highest among neurologists in the outpatient group and among psychiatrists in the inpatient group. Implementing reminders substantially improved the screening rates across outpatient specialties, particularly in neurology and neurosurgery. This increase in inpatient psychiatry screening rates, despite the lack of direct reminders, may be attributable to spillover effects from the outpatient intervention. One of our hospital branches is a psychiatric hospital, which may have reinforced this behavior.

This study has several limitations. First, because data were collected from a single hospital, some patients may have received HLA-B*15:02 testing at other institutions before referral to our hospital, and these external results may not have been recorded in our database. Such cases could have been misclassified as unscreened, potentially leading to an underestimation of screening rates. To solve this problem, 2011 data were used to refine the definition of a new user. Second, the first year of data collection (2012) may not be fully representative of CBZ prescription trends, although the overall decline in CBZ use over time is consistent with broader trends in Taiwan. Third, given the retrospective nature of this study, causal inference is limited. No formal concurrent institutional campaigns were implemented during the intervention period; however, we cannot rule out the potential influence of informal physician education, peer interactions, or national discourse on pharmacogenomic screening as unmeasured confounders. Although there is an observed association between CPOE reminders and increased screening, future prospective studies are needed to confirm these findings and establish causal relationships. Fourth, the relatively small sample size in the post-intervention outpatient group (n = 54) and the imbalance between outpatient and inpatient cohorts (1324 vs. 287) limit the statistical power of subgroup analyses and may reduce the precision of between-group comparisons. While the overall effect remained significant, wider confidence intervals in some analyses underscore the need for larger future studies to confirm these findings and allow for finer stratification. Finally, while the intervention improved screening rates, we did not assess downstream clinical outcomes such as the incidence of SCARs or CBZ-related hospitalizations. Given the rarity of such events and the limited follow-up time, future studies should incorporate longer observation periods and clinical outcomes to evaluate patient-level safety benefits.

In addition, future studies should incorporate qualitative or survey-based methodologies to explore underlying reasons for suboptimal screening rates, including physician decision-making processes and patient-related barriers such as blood draw concerns or wait times.

## 5. Conclusions

Despite the decline in CBZ use, HLA-B*15:02 genetic testing remains an essential component in ensuring patient safety. This study demonstrated that integrating a screening reminder into a CPOE system significantly improved the screening rates of HLA-B*15:02 in outpatient settings, underscoring the importance of CPOE reminders as a practical and effective tool for enhancing adherence to pharmacogenomic guidelines.

Expanding the aforementioned reminder system to inpatient CPOE workflows may further improve screening rates and mitigate the risk of SCARs among patients receiving CBZ. Future research should include patients from multiple centers to validate these findings and explore the long-term effect of CPOE interventions on clinical outcomes and the broader implementation of precision medicine.

This study illustrates the significant effect of computerized physician order entry (CPOE) reminders on increasing the screening rate for the HLA-B*15:02 allele prior to prescribing carbamazepine (CBZ). By integrating these reminders into clinical workflows, healthcare providers can proactively identify patients at risk of severe cutaneous adverse reactions, leading to more informed treatment decisions and improved patient safety. These findings highlight the vital role of informatics in promoting the implementation of pharmacogenomic guidelines and enhancing the quality of patient care.

## Figures and Tables

**Table 1:** Patient characteristics in outpatient and inpatient groups.

	Outpatient Group (n = 1324)	Inpatient Group (n = 287)	
Sex			*p* = 0.031
Male	563 (42.5%)	142 (49.5%)	
Female	761 (57.5%)	145 (50.5%)	
Age, years			
Mean (SD)	55.5 ± 18.3	55.9 ± 18.9	*p* = 0.794
≤40	320 (24.2%)	75 (26.1%)	*p* = 0.362
41–60	444 (33.5%)	89 (31.0%)	
61–80	449 (33.9%)	91 (31.7%)	
>80	111 (8.4%)	32 (11.1%)	
Physicians’ specialty			*p* < 0.001
Neurology	417 (31.5%)	22 (7.7%)	
Psychiatry	257 (19.4%)	109 (38.0%)	
Neurosurgery	157 (11.9%)	10 (3.5%)	
Others	493 (37.2%)	146 (50.9%)	
Treatment indication *			*p* < 0.001
Trigeminal neuralgia	405 (30.6%)	4 (1.4%)	
Bipolar disorder	146 (11.0%)	38 (13.2%)	
Epilepsy	148 (11.2%)	22 (7.7%)	
Off-label use **	625 (47.2%)	223 (77.7%)	

* Treatment indications were defined using International Classification of Diseases, Ninth Revision, Clinical Modification (ICD-9-CM) and International Classification of Diseases, Tenth Revision, Clinical Modification/Procedure Coding System (ICD-10-CM/PCS) codes. ** Off-label use was defined as not being used to treat trigeminal neuralgia, bipolar disorder, or epilepsy.

**Table 2:** Comparison of HLA-B*15:02 screening rates before and after the intervention in the outpatient and inpatient groups.

	Before	After	OR (95% CI)	*p* Value
Outpatient (Intervention)	301/1270 (23.7%)	30/54 (55.6%)	4.02 (2.31–6.98)	*p* < 0.001
Inpatient (Control)	84/262 (32.1%)	11/25 (44.0%)	1.67 (0.72–3.89)	*p* = 0.225

**Table 3:** Factors associated with HLA-B*15:02 screening rates in outpatients, stratified by age group.

Age	Before	After	*p* Value
<40	93/305 (30.5%)	7/15 (46.7%)	*p* = 0.187
41–60	96/427 (22.5%)	9/17 (52.9%)	*p* = 0.004
61–80	93/431 (21.6%)	12/18 (66.7%)	*p* < 0.001
>80	19/107 (17.8%)	2/4 (50%)	*p* = 0.106

**Table 4:** Factors associated with HLA-B*15:02 screening rates in inpatients, stratified by age group.

Age	Before	After	*p* Value
<40	37/67 (55.2%)	7/8 (87.5%)	*p* = 0.080
41–60	23/81 (28.4%)	3/8 (37.5%)	*p* = 0.589
61–80	19/84 (22.6%)	1/7 (14.3%)	*p* = 0.609
>80	5/30 (16.7%)	0/2 (0.0%)	*p* = 0.530

**Table 5:** Factors associated with HLA-B*15:02 screening rates of outpatients, stratified by physician specialty.

Specialty	Before	After	*p* Value
Neurology	175/390 (44.9%)	22/27 (81.5%)	*p* < 0.001
Neurosurgery	10/155 (6.5%)	2/2 (100.0%)	*p* < 0.001
Psychiatry	98/255 (38.4%)	2/2 (100.0%)	*p* = 0.075
Others	18/470 (3.8%)	4/23 (17.4%)	*p* = 0.002

**Table 6:** Factors associated with HLA-B*15:02 screening rates of inpatients, stratified by physician specialty.

Specialty	Before	After	*p* Value
Neurology	9/20 (45.0%)	0/2 (0.0%)	*p* = 0.217
Neurosurgery	1/10 (10.0%)	0/0	not applicable
Psychiatry	59/98 (60.2%)	11/11 (100.0%)	*p* = 0.009
Others	15/134 (11.2%)	0/12 (0.0%)	*p* = 0.221

**Table 7:** Multivariable logistic regression for predictors of HLA-B*15:02 screening.

Variable	Adjusted OR (95% CI)	*p* Value
Intervention (post vs. pre)	1.58 (0.57–4.41)	0.260
Outpatient vs. inpatient	0.33 (0.23–0.48)	<0.001
Interaction (post × outpatient)	4.20 (1.20–14.74)	0.021
Male (ref: female)	0.80 (0.61–1.05)	0.090
Age (per year)	0.99 (0.98–1.00)	0.685
Diagnosis (ref: off-label use)		
Epilepsy	0.31 (0.18–0.52)	<0.001
Trigeminal neuralgia	1.83 (1.27–2.64)	0.001
Bipolar disorder	2.57 (1.69–3.93)	<0.001
Physician (ref: others)		
Neurology	21.40 (13.88–33.01)	<0.001
Neurosurgery	1.52 (0.77–3.03)	0.216
Psychiatry	11.97 (7.61–18.82)	<0.001

OR: Odds ratio; CI: confidence interval. Logistic regression adjusted for sex, age, physician specialty, and diagnosis. The interaction term indicates effect modification by clinical setting.

## Data Availability

The data presented in this study are available on request from the corresponding authors.

## Abbreviations

The following abbreviations are used in this manuscript:

CBZ	Carbamazepine
SCARs	severe cutaneous adverse reactions
SJS	Stevens–Johnson syndrome
TEN	toxic epidermal necrolysis
CPOE	computerized physician order entry
